# Trends in tuberculosis mortality among older adults in China, 2004–2021: a Joinpoint regression and age–period–cohort analysis

**DOI:** 10.3389/fpubh.2024.1500539

**Published:** 2025-01-06

**Authors:** Mengdi Zhang, Xin Wang, Yiran Xiao, Qiqi Wang, Fei Huang, Xiang Ren, Xiaomin Guo, Wenshan Sun, Jinqi Deng, Qi Jiang, Jianjun Liu, Wenjing Zheng, Hongyan Yao

**Affiliations:** ^1^Office of Education and Training (Graduate School), Chinese Center for Disease Control and Prevention, Beijing, China; ^2^Office of Epidemiology (Technical Guidance Office for Patriotic Health Work), Chinese Center for Disease Control and Prevention, Beijing, China; ^3^Center for Tuberculosis Control and Prevention, Chinese Center for Disease Control and Prevention, Beijing, China; ^4^Division of Infectious Diseases, Chinese Center for Disease Control and Prevention, Beijing, China; ^5^Center for Logistics Management and Operations, Chinese Center for Disease Control and Prevention, Beijing, China; ^6^Office of Finance, Chinese Center for Disease Control and Prevention, Beijing, China; ^7^Chinese Antituberculosis Association, Beijing, China

**Keywords:** tuberculosis, mortality, older adult, Joinpoint regression, age-period-cohort analysis

## Abstract

**Background:**

Tuberculosis (TB) remains a major public health problem in China and globally, particularly among older adults. This study aimed to examine secular trends in TB mortality among older adults in China and the net effects of age, period, and cohort.

**Methods:**

Data from the National Disease Surveillance Points (DSPs) system were analyzed using Joinpoint regression to determine annual changes in TB mortality among individuals aged 60 years and older from 2004 to 2021. An age–period–cohort (APC) analysis using the intrinsic estimator (IE) method was conducted to estimate the independent effects of age, period, and cohort.

**Results:**

The age-standardized TB mortality rate was 5.68 per 100,000, with higher rates observed in men, rural areas, and western regions. TB mortality among older adults declined overall from 2004 to 2021, although the rate of decline has slowed in recent years. The APC analysis revealed increased TB mortality with age, with the relative risk (RR) rising from 0.57 in the 60–64 age group to 1.53 in the 80–84 age group. The period effect decreased from 2007 to 2021, showing a higher risk effect in rural areas (RR = 1.51) than in urban areas (RR = 1.16) during 2007–2011, but this trend reversed in the period 2017–2021. The cohort effect generally declined, with the exception of certain demographic groups that showed an increase in the 1952–1956 and 1957–1961 birth cohorts.

**Conclusion:**

TB mortality among older adults in China decreased from 2004 to 2021, although the decline has slowed in recent years. Variations in age, period, and cohort effects highlight differences by gender, urban and rural areas, and regions, providing insights for targeted intervention strategies.

## 1 Introduction

Tuberculosis (TB), a chronic infectious disease caused by *Mycobacterium tuberculosis*, is one of the major infectious diseases in the world. The World Health Organization (WHO) reported that in 2023, an estimated 10.8 million people developed TB, and 1.3 million people died from the disease (1). In China, TB poses a serious threat to public health and is classified as a category II notifiable disease. Despite ongoing efforts and progress, China still faces one of the highest TB burdens globally, ranking third in the world on several TB burden indicators (2). Additionally, in 2023, a total of 741,000 new TB cases and 25,000 deaths were reported in China, with an incidence of 52.0 per 100,000 and a mortality of 2.0 per 100,000 (1).

It is widely acknowledged that older adults are particularly susceptible to TB diseases (3–6). Rising life expectancy and declining fertility rates have accelerated the aging of the population (7). The characteristics of older adults, such as weakened immunity, malnutrition due to poverty or illness, comorbidities (such as diabetes), and poor access to health services, have made TB in older adults a major problem in many countries (8, 9). According to estimates from the WHO, the proportion of the population aged 60 years and older in China is expected to increase from 12.4% in 2010 to 28.0% in 2040 (10). The reported incidence of TB among older adults in China was 2 to 3 times higher than that in younger populations (11), with those aged 60 and older being a high-risk group for TB mortality (12). Building a TB-free world is a goal of the WHO End TB Strategy. Its key targets include a 75% reduction in TB deaths and a 50% reduction in TB incidence by 2025, and a 90% reduction in deaths and an 80% reduction in incidence by 2030, compared to 2015 (13). China faces significant challenges in reaching the goals of the WHO End TB Strategy by 2030 (14). Identifying the distribution of TB and high-risk groups in the older adult population is crucial for TB prevention and control strategies in China.

Therefore, this study utilized Joinpoint regression to describe the trends in TB mortality among older adults in China. Unlike previous TB mortality studies in China among all age groups (12, 15), this study focused only on the older adult population. It analyzed differences by gender, residence, and region—factors that have been overlooked in previous studies. Furthermore, this study aimed to estimate age, period, and cohort effects on TB mortality to identify high-risk groups among older adults in China using an age–period–cohort (APC) model.

## 2 Materials and methods

### 2.1 Data source

Data on TB mortality among people aged 60 years and older in China were obtained from the National Disease Surveillance Points (DSPs) system (16). This population-based death registration system initially collected data only from “unrepresentative surveillance points,” then gradually expanded to 161 surveillance points across 31 provinces in 2004 and 605 points in 2013, covering 323.8 million people (24.3% of the country’s total population). TB was defined according to the International Classification of Diseases, 10th Revision, codes A15–A19 or B90.

This study used aggregate annual mortality from 2004 to 2021, grouped by sex (male and female subjects), age (60–64, 65–69, 70–74, 75–79, 80–84, and 80+), residence (urban and rural areas), and region (eastern, central, and western). All counties (including county-level cities) were defined as rural, and all districts were defined as urban. The three regions were divided according to the National Bureau of Statistics: the eastern region of China included Beijing, Tianjin, Shanghai, Hebei, Liaoning, Jiangsu, Zhejiang, Fujian, Shandong, Guangdong, and Hainan; the central region included Shanxi, Jilin, Heilongjiang, Anhui, Jiangxi, Henan, Hubei, and Hunan; and the western region included Chongqing, Sichuan, Guizhou, Yunnan, Shaanxi, Gansu, Qinghai, Inner Mongolia, Guangxi, Xizang, Ningxia, and Xinjiang. The 2010 Chinese census was used to determine the age-standardized mortality rate (ASMR).

### 2.2 Statistical analysis

#### 2.2.1 Joinpoint regression

The Joinpoint regression model (17) was used to evaluate the time trends in TB mortality among people aged 60 years and older in China from 2004 to 2021. This model can divide the longitudinal variations into different segments using piecewise regression and identify the segment trends that are statistically significant. Regression fitting was performed on the natural logarithm of the mortality rate in different segments, and then the annual percentage change and its 95% confidence interval (CI) were calculated for each period (18). The annual percentage change and the average annual percentage change were the main indicators used to describe the temporal variation in the Joinpoint regression model. They were considered statistically significant when compared to 0 with *p*-values of <0.05. Joinpoint (version 5.0.2; National Cancer Institute, Calverton, MD, USA) was used to create this model.

#### 2.2.2 Age–period–cohort analysis

An age–period–cohort (APC) model (19) was applied to assess the impact of age, period, and cohort effects on TB mortality among older adults in China from 2007 to 2021. The APC model has been commonly used in sociology and epidemiology for several decades. However, when the APC model was first proposed, it was difficult to calculate the specific effects of age, period, and cohort due to the linear correlation among these variables (cohort = period-age), a situation known as the non-identification problem (20). To solve this problem, Yang and Fu introduced the intrinsic estimator (IE) method, which effectively distinguishes the effects of age, period, and cohort and provides unbiased and relatively efficient estimation results (21).

In this study, the age effect refers to the differences in TB mortality across age groups caused by aging-related factors. The period effect refers to the influence of human factors on TB mortality, such as medical technology. The cohort effect refers to the change in TB mortality due to different exposures to risk factors among people of different birth years.

In the APC model with the IE method, equal intervals were required for age, period, and cohort, and TB mortality was calculated for each 5-year age group (60–64, 65–69, 70–74, 75–79, and 80–84). Hence, the period and cohort were divided into 5-year intervals: three periods (2007–2011, 2012–2016, and 2017–2021), and seven cohorts (1927–1931, 1932–1936, 1937–1941, 1942–1946, 1947–1951, 1952–1956, and 1957–1961). In particular, data for the 80+ age group or the years 2004–2006 were excluded from the APC analysis considering the model conditions. Due to the increase in the number of monitoring centers from 161 to 605 in 2013, the number of TB deaths in different years was weighted differently between 2012 and 2013-2016. The average TB mortality from 2013-2016 was used to represent TB mortality for the period 2012-2016 in the study. Since neighboring birth cohorts partially overlap, the birth cohorts are usually described by their middle year (22). For subjects aged 75–79 and 80–84 years from 2007 to 2011, their birth cohorts were from 1928 to 1936 and from 1923 to 1931, respectively. They were denoted as from 1932 to 1936 and from 1927 to 1931, respectively. The APC model could be expressed as follows (12):


$$Y=\log\left(R\right)=\mu_{0}+\alpha \times \mathrm{ag}e_{A}+\beta \times \mathrm{perio}d_{P}+\gamma \times \mathrm{cohor}t_{C}+\varepsilon$$

where R is the TB mortality of different groups; α, β, and γ denote the coefficients of age, period, and cohort of the APC model; μ_0_ and *ε* represent the intercept item and residual. These model coefficients (α, β, γ) were used to calculate the exponential value (e^coef.^), which denotes the relative risk (RR) of mortality of a particular age, period, or birth cohort relative to each average level. The analyses presented in this study were conducted using the APCG1 package in the R software (version 3.5.0; The R Foundation for Statistical Computing, Vienna, Austria).

## 3 Results

### 3.1 Joinpoint regression analysis of TB mortality

Trends in the crude mortality rate (CMR) and age-standardized mortality rate (ASMR) among older adults by gender, urban and rural areas, and regions from 2004 to 2021 are shown in Table 1. In 2021, there were 3,240 TB deaths among the approximately 55 million surveilled population aged 60 years and older, including 2,420 male and 820 female patients. The CMR of TB in older adults was 5.90 per 100,000, while the ASMR of TB was 5.68 per 100,000. Higher rates were observed in males (9.15 per 100,000) than in females (2.65 per 100,000), in rural areas (6.18 per 100,000) than in urban areas (4.70 per 100,000), and in western regions (9.87 per 100,000) than in central (5.12 per 100,000) and eastern (3.73 per 100,000) regions. The ASMR showed a significant decline, with an average annual percentage change (AAPC) of −10.00% (*p* < 0.001), decreasing significantly across gender, urban vs. rural, and regional categories from 2004 to 2021. The largest reduction in ASMR was observed in the female group, with an AAPC of −11.32% (*p* < 0.001), while the smallest reduction was observed in urban areas, with an AAPC of −8.30% (p < 0.001).

**Table 1:** Variation in TB mortality among people aged 60 years and older by gender, urban and rural areas, and regions in China (per 100,000), 2004–2021.

Year	Male		Female		Urban		Rural		Eastern		Central		Western		Overall	
	CMR	ASMR	CMR	ASMR	CMR	ASMR	CMR	ASMR	CMR	ASMR	CMR	ASMR	CMR	ASMR	CMR	ASMR
2004	45.48	47.60	20.53	20.15	18.60	19.09	40.75	40.85	18.42	18.07	30.68	31.90	56.98	57.71	32.68	32.92
2005	49.01	51.23	22.11	21.55	19.35	19.66	44.35	44.51	19.97	19.48	33.40	34.78	61.07	62.28	35.21	35.44
2006	31.14	32.44	12.04	11.77	12.91	13.07	26.13	26.13	16.13	15.69	20.96	21.81	30.31	30.46	21.32	21.36
2007	27.80	29.08	10.39	10.13	11.80	11.77	23.16	23.27	14.25	13.78	16.89	17.52	28.88	29.18	18.85	18.90
2008	25.85	26.64	8.93	8.68	10.12	9.96	21.87	21.91	12.89	12.44	14.97	15.25	26.95	27.48	17.19	17.13
2009	24.66	26.79	8.23	8.31	9.73	9.92	20.51	21.47	11.65	11.68	13.80	14.76	26.70	28.06	16.18	16.74
2010	21.98	25.20	8.56	8.84	9.77	10.59	18.87	20.22	9.61	10.03	12.03	13.24	27.97	30.82	15.05	16.16
2011	20.36	22.95	7.57	7.63	8.60	9.62	17.02	17.50	7.49	7.79	10.44	10.97	28.70	30.86	13.65	14.31
2012	18.87	19.52	8.02	7.85	7.97	7.77	16.66	16.72	7.45	7.12	9.47	9.66	28.18	28.57	13.24	13.19
2013	15.93	16.51	5.71	5.58	8.26	8.11	11.85	12.02	7.00	6.88	9.17	9.43	19.44	19.53	10.71	10.77
2014	14.64	15.08	4.75	4.61	7.97	7.85	10.34	10.42	6.50	6.33	9.16	9.35	15.70	15.73	9.59	9.61
2015	14.58	15.07	5.31	5.19	8.28	8.15	10.58	10.69	7.10	6.92	8.29	8.43	16.34	16.40	9.85	9.86
2016	13.75	13.86	5.13	4.85	7.94	7.62	9.98	9.81	6.69	6.31	7.26	7.17	16.46	16.23	9.33	9.09
2017	13.39	13.45	4.49	4.24	7.16	6.91	9.62	9.43	6.32	5.92	6.86	6.73	16.14	16.04	8.82	8.59
2018	12.88	12.81	4.68	4.35	6.95	6.61	9.53	9.23	6.21	5.73	6.53	6.34	16.14	15.90	8.67	8.34
2019	11.71	12.53	3.79	3.82	6.44	6.53	8.25	8.61	5.48	5.50	6.41	6.76	13.01	13.70	7.63	7.89
2020	10.40	10.88	3.12	3.04	5.57	5.45	7.22	7.34	5.00	4.86	5.72	5.84	10.68	10.93	6.63	6.67
2021	9.16	9.15	2.87	2.65	4.99	4.70	6.36	6.18	3.94	3.73	5.29	5.12	10.20	9.87	5.90	5.68
AAPC (%)	−9.16	−8.69	−11.18	−11.32	−7.86	−8.30	−9.97	−9.93	−9.12	−9.24	−10.33	−10.81	−8.86	−9.01	−8.189	−10.00
*t*	−8.760	−9.621	−6.220	−6.332	−6.987	−6.020	−9.409	−10.401	−8.668	−8.206	−11.807	−9,491	−12.468	−12.447	−11.326	−7.355
*p*	<0.001	<0.001	<0.001	<0.001	<0.001	<0.001	<0.001	<0.001	<0.001	<0.001	<0.001	<0.001	<0.001	<0.001	<0.001	<0.001

*CMR: crude mortality rate, ASMR: age-standardized mortality rate, AAPC: annual average percentage change.

Table 2 illustrates the temporal variation in age-specific TB mortality from 2004 to 2021. There were significant downward trends in all age groups of older adults. The largest reductions were observed in the 65–69 and 60–64 age groups, with AAPCs of −11.33% (*p* < 0.001) and − 10.56% (p < 0.001), respectively.

**Table 2:** Age-specific TB mortality variation over time (per 100,000), 2004–2021.

Year	60–64	65–69	70–74	75–79	80–84	85–89
2004	17.66	27.14	38.34	46.74	62.49	63.22
2005	18.51	27.42	42.68	54.10	60.20	76.11
2006	11.64	16.55	27.36	30.91	36.21	40.34
2007	10.21	14.44	21.15	27.75	39.45	36.39
2008	8.56	13.55	20.39	28.12	30.34	30.71
2009	7.03	13.07	18.44	25.05	31.73	51.63
2010	6.90	12.30	15.74	22.34	32.31	62.45
2011	7.72	10.08	14.02	20.82	26.57	47.41
2012	6.88	10.06	15.08	21.34	23.26	27.32
2013	5.12	9.02	12.92	15.89	19.48	23.00
2014	4.38	7.76	11.42	15.45	18.21	18.60
2015	4.48	8.20	10.75	15.59	18.64	23.22
2016	4.69	7.10	9.61	13.48	17.29	23.33
2017	4.69	7.04	8.92	12.11	16.09	21.31
2018	4.54	6.61	8.81	11.60	14.97	23.11
2019	3.08	5.28	8.61	13.39	15.37	25.58
2020	2.96	4.52	6.90	10.75	14.56	19.14
2021	2.58	3.87	5.99	9.12	12.04	16.02
AAPC (%)	−10.56	−11.33	−10.54	−8.74	−8.51	−6.93
*t*	−5.857	−7.696	−9.335	−14.536	−9.682	−6.534
*p*	<0.001	<0.001	<0.001	<0.001	<0.001	<0.001

### 3.2 The variation of age, period, and cohort on TB mortality

**Figure 1:**
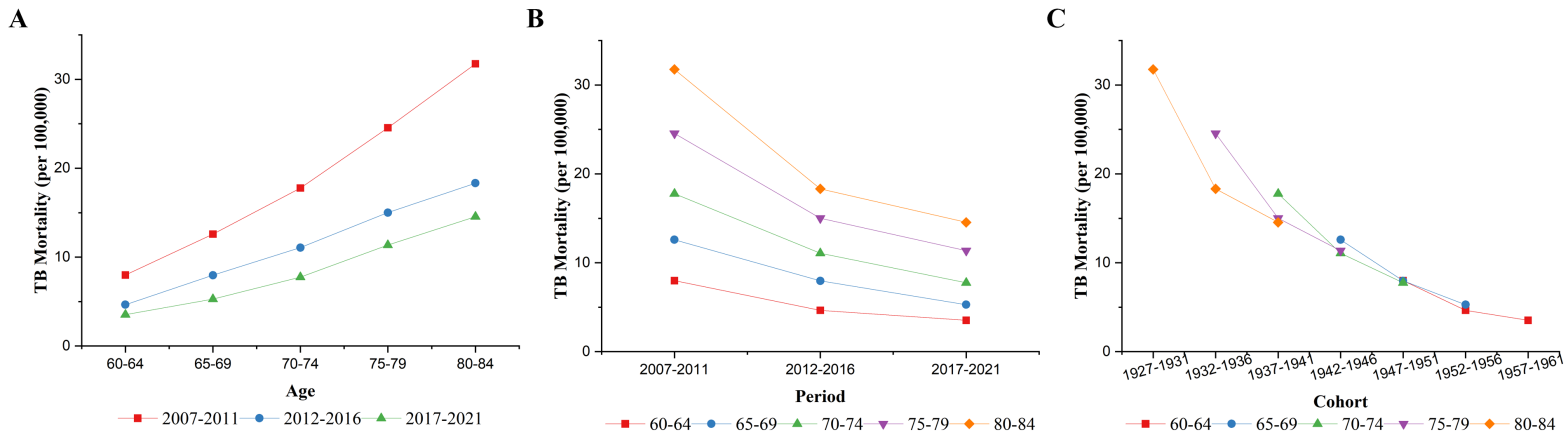
Variations in TB mortality by **(A)** age, **(B)** period, and **(C)** birth cohort.

Figure 1 shows the changes in TB mortality by age, period, and cohort from 2007 to 2021. As shown in Figure 1A, TB mortality among older adults in China increased with age in all three time periods: 2007–2011, 2012–2016, and 2017–2021. Figure 1B shows the trends in TB mortality for different age groups from 2007 to 2021. TB mortality decreased in all age groups from 2007 to 2021, which is consistent with the results shown in Table 2. The downward trends were more pronounced before the 2012–2016 period than after it. Figure 1C shows the cohort-based variation in age-specific TB mortality, showing that the later birth cohorts all had lower mortality than the earlier cohorts in the same age group. The decline in TB mortality was more moderate in later birth cohorts than in earlier cohorts.

### 3.3 Age–period–cohort analysis of TB mortality

#### 3.3.1 Age effect

**Figure 2:**
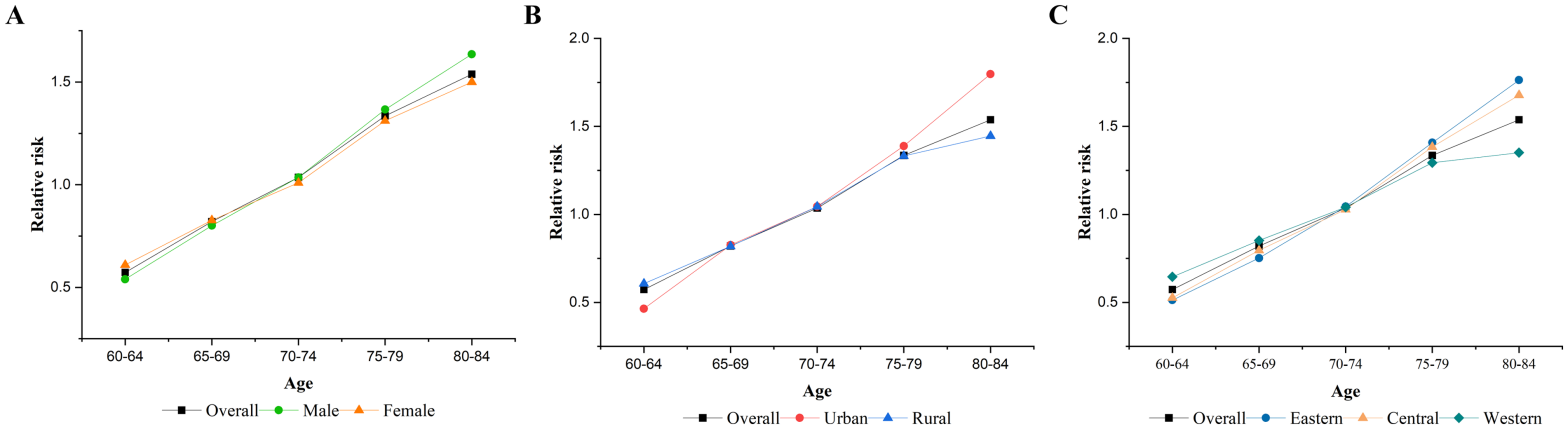
Age effects on TB mortality. **(A)** Gender, **(B)** urban–rural, **(C)** eastern, central, and western.

After controlling for period and cohort effects, the age effect on TB mortality among older adults in China is shown in Figure 2. The risk of dying from TB increased with age, as the RR increased from 0.57 in the 60–64 age group to 1.54 in the 80–84 age group. The age effects, stratified by gender, urban and rural areas, and regions, also showed upward trends similar to the overall effect. However, the increase was faster for men than women, in urban areas than in rural areas, and the eastern regions than in the central and western regions.

#### 3.3.2 Period effect

**Figure 3:**
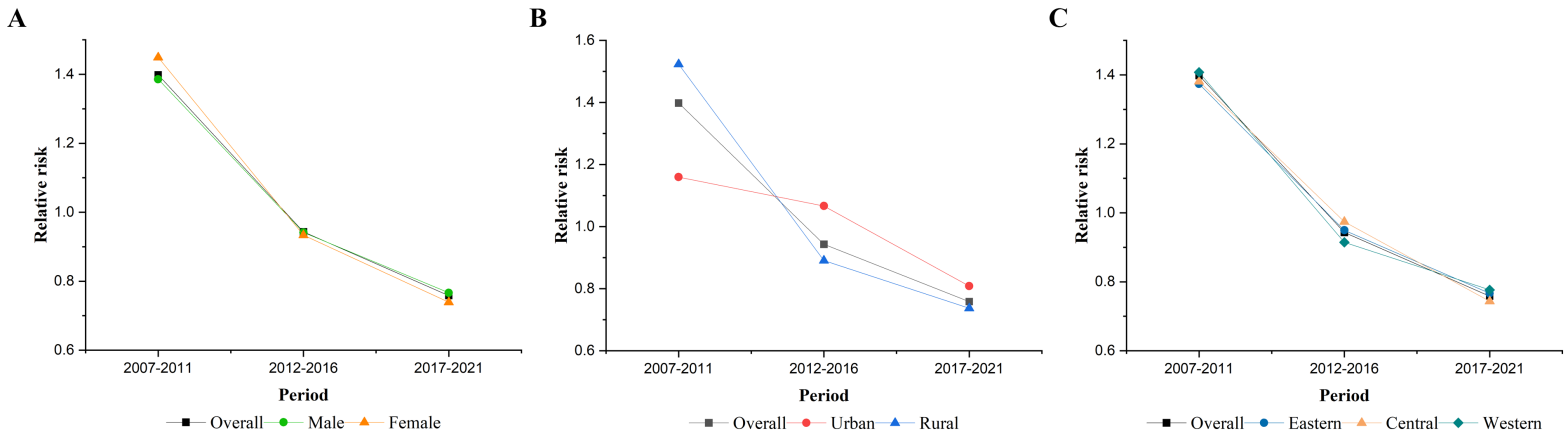
Period effects on TB mortality. **(A)** Gender, **(B)** urban–rural, and **(C)** eastern, central, and western.

Figure 3 shows that after controlling for age and cohort effects, the RR of the period effect on TB mortality among older adults in China continuously decreased from 2007 to 2021, with the highest risk occurring from 2007 to 2011 (RR = 1.40, 95% CI: 1.36–1.43) and the lowest risk occurring from 2017 to 2021 (RR = 0.76, 95% CI: 0.74–0.78). After stratification, the period effects in different gender and region groups were similar to the overall effect. As shown in Figure 3B, the risk effect was higher in rural areas (RR = 1.52, 95% CI: 1.49–1.56) than in urban areas (RR = 1.16, 95% CI: 1.06–1.26) for the period 2007–2011, while it was higher in urban areas (RR = 0.81, 95% CI: 0.74–0.89) than in rural areas (RR = 0.74, 95% CI: 0.72–0.76) for the period 2017–2021.

#### 3.3.3 Cohort effect

**Figure 4:**
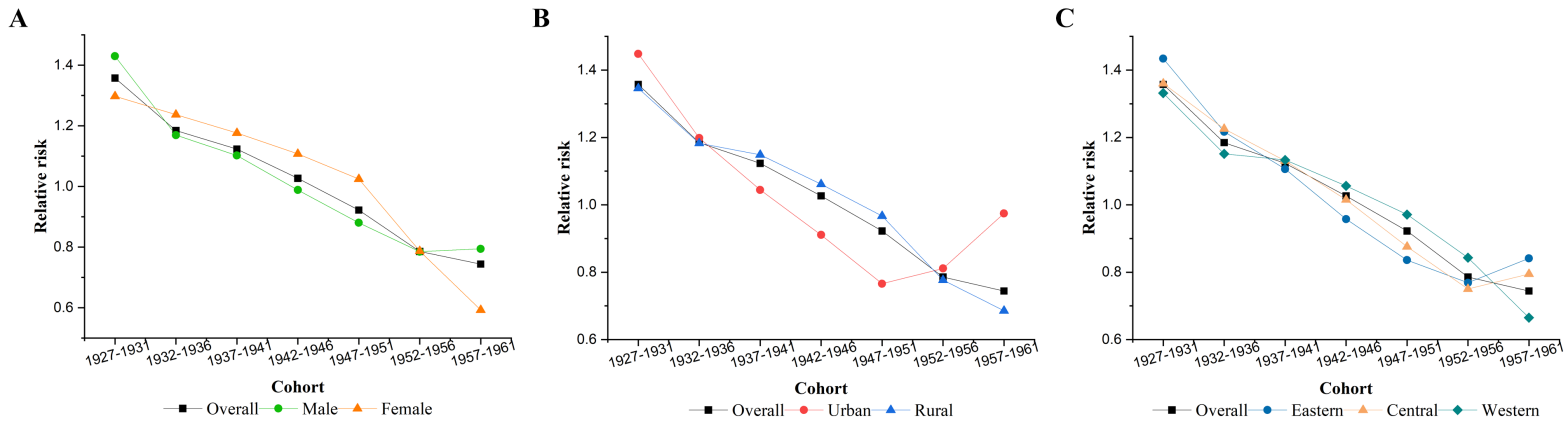
Cohort effects on TB mortality. **(A)** Gender, **(B)** urban–rural, **(C)** eastern, central, and western.

After controlling for age and period effects, Figure 4 illustrates the cohort effect, indicating that individuals born in later cohorts had a lower RR of TB mortality. The cohort with the highest RR of TB mortality was the 1927–1931 cohort (RR = 1.36, 95% CI: 1.29–1.43), and the RR decreased to the lowest value for the 1957–1961 cohort (RR = 0.74, 95% CI: 0.69–0.80). The cohort effect for those born after 1947 was no longer higher than the overall average. Stratified analyses revealed that the cohort effects for women, rural areas, and western regions also followed an overall downward trend. In contrast, the risk for the male, eastern, and central China groups rebounded after being minimized in the 1952–1956 cohort. Similarly, in urban areas, the RR of the cohort effect decreased from the 1927–1931 cohort, reaching its lowest value in the 1947–1951 cohort (RR = 0.77, 95% CI: 0.67–0.88), before increasing again in the 1952-1956 and 1957-1961 cohort.

## 4 Discussion

This study revealed a statistically significant decline in TB mortality among older adults in China from 2004 to 2021, which is consistent with other studies in China (12) and globally (1, 23). Economic prosperity, leading to improved socioeconomic conditions and living standards (such as better housing and nutrition), contributes to the continued decline in mortality (24). The decline in TB mortality has also benefited from a series of interventions and policies that have been put in place by the government. For instance, China has actively promoted disease control efforts, achieving full implementation of the DOTS strategy around 2004 (25). Since then, TB mortality has declined at a slower rate in recent years, a trend that has also been observed in TB incidence (22). Previous studies found that TB mortality increased following the COVID-19 outbreak (26, 27). TB–COVID-19 co-infection was associated with an elevated risk of unfavorable clinical outcomes and higher rates of mortality (28). However, the upward trend was not observed following the outbreak in our study. We found that TB mortality among older adults in China decreased in 2020 and 2021. This observation was consistent with the findings presented by Zhang et al. Their study found that mortality decreased immediately at the start of the COVID-19 pandemic, then increased sharply after January 2022, which resulted in a long-term upward trend during the pandemic (29). Due to limitations in available data resources, we could not analyze the TB mortality trends for 2022 and beyond. The long-term impact of the COVID-19 pandemic on TB mortality will be examined in subsequent research.

The study reported variations in TB mortality across different gender and age groups, residences, and regions. TB mortality was higher among males, in rural areas, and in the western region, which is consistent with previous studies (15, 30–33). The higher TB mortality among males may be attributed to factors such as increased participation in social activities (34), high labor intensity (34), excessive smoking (35) and alcohol consumption (36), poor resistance (37), and incomplete antituberculosis treatment (34). The higher mortality in rural areas and the western region may be due to the lower levels of economic development and limited access to healthcare services (38). Patients with higher socioeconomic status are more likely to receive superior treatment and additional diagnostic procedures (39). Furthermore, TB is associated with education level and lifestyle factors (11, 40). The level of education in rural areas is lower than in urban areas due to geographical location and hukou policies in China (41). In terms of personal lifestyle, rural residents have a less healthy lifestyle than urban residents in terms of smoking, drinking, and social interactions (42). We also found different decline rates in TB mortality after stratification by gender, residence, and region.

Given the intricate interactions among age, period, and cohort factors, we applied the age–period–cohort model and the IE algorithm to quantify their net effects on TB mortality. The age effect on TB mortality increased with advancing age. The higher TB mortality among older people may be due to waning immunity and increased comorbidities (43). Additionally, older people usually experience a worse prognosis and are often under-treated (44). Our study showed that compared to the female group, aging may have a greater impact on increasing the RR of TB mortality in men. A similar finding for TB incidence was reported by Li (45). Furthermore, this study found that aging increased the RR of TB mortality in urban areas more than in rural areas, and the eastern regions more than in the central and western regions. This was in contrast to the order of TB mortality among the different groups, but the results were not contradictory. This phenomenon can be attributed to the higher incidence of TB deaths in rural areas and the western regions occurring in relatively younger age groups. Lower economic and educational levels, poor nutrition, and insufficient health knowledge affect the survival time of TB patients (46).

The period effect may be influenced by various environmental, historical, and economic factors. The age–period–cohort analysis revealed a decline in TB mortality over time, which is consistent with the results of the Joinpoint regression model and previous results (33, 47). Intriguingly, the rate of decline was more pronounced in rural areas than in urban areas, which may be attributed to fast-paced economic growth, enhanced healthcare coverage (especially in rural areas), and rigorous respiratory TB control policies (15). This finding was contrary to the urban–rural difference in TB incidence. Some studies found that the incidence of TB decreased more significantly in urban than in rural areas in recent years (22, 48). Moreover, the downward trend after 2012–2016 was not as pronounced as it was before 2012–2016, which reminded us that more attention should be paid to innovative research.

The cohort effect reflected influencing factors that arise earlier in the life course and accumulate over time. The cohort effect on TB mortality showed a continuously decreasing trend from the 1927–1931 birth cohort to the 1957–1961 birth cohort, with the exception of certain demographic groups, where the risk tended to increase in the last one or two birth cohorts. The increase in TB mortality among individuals from the 1952–1956 birth cohort and the 1957–1961 birth cohorts possibly was related to their early-life exposure to the Chinese famine (49). Malnutrition has been identified as a significant risk factor for TB (50, 51), with one study suggesting that prenatal and early-life exposure to malnutrition may increase the risk of TB in the exposed generation and their offspring (52). Several factors may contribute to the phenomenon. Maternal and postnatal malnutrition is known to change lung architecture and compromise immunological development, increasing morbidity or mortality from various infectious diseases, including TB (53). Additionally, inherited epigenetic changes were affected by prenatal exposure to famine and may have contributed to increased susceptibility in their offspring (54). Research has also found that individuals conceived during famine periods were more likely to prefer fatty foods and engage in less physical activity. Their children were often raised with the same lifestyle habits, which may result in immunologic changes that increase the risk of TB infection and its progression (52).

The strength of our study is the nationwide nature of the data and further analysis stratified by gender, urban and rural, and regional categories. The difference between this study and the previous national study (12) was that we used data on TB mortality from the National Disease Surveillance Points (DSPs) system instead of the National Notifiable Disease Reporting System (NNDRS) (55). TB mortality from the NNDRS reflects patients who received treatment instead of all patients and is defined as death during treatment from any cause. TB mortality in the DSPs is determined by the physician, as the cause of death is from TB. However, this study also has several limitations. First, the under-reporting of TB deaths should be noted. The data in the DSPs were collected from routine surveillance records, and the number of TB deaths in China may be higher than reported. However, the DSPs system is the only national mortality surveillance system that covers all causes of death in China. The system has expanded its surveillance population from 6 to 24% of the Chinese population since 2013, covering almost a quarter of the population. It is the only feasible option for obtaining reliable and valid information on TB mortality in the country. Second, the increase in the number of monitoring centers from 161 to 605 in 2013 may cause some inconsistency in data collection. Although we used the average of the last 4 years to replace the data for 2012 when calculating TB mortality for the period 2012–2016, the findings of this study should still be treated with caution. Third, in addition to gender, urban and rural areas, and regions (eastern, central, and western), other factors, such as economic level and comorbidities (such as HIV and diabetes) were not included in the analysis. These factors would also influence the epidemic status and control of TB and should be considered in future studies.

## 5 Conclusion

The overall TB mortality among older adults in China declined from 2004 to 2021, but the extent of the reduction varied among different demographic groups. The age effect on TB mortality presented a significantly increasing trend in older people. Period effects declined, with smaller decreases in recent years. Further efforts should be made to improve the surveillance, diagnosis, treatment, and management of the disease. The rate of decline was more pronounced in rural areas than in urban areas. Birth cohorts exhibited a general decline in TB mortality, although there were upward trends observed in the most recent 1952–1956 and 1957–1961 birth cohorts.

## Data Availability

The original contributions presented in the study are included in the article/supplementary material, further inquiries can be directed to the corresponding authors.
